# Revealing low-dose radiation damage using single-crystal spectroscopy

**DOI:** 10.1107/S0909049511004250

**Published:** 2011-03-19

**Authors:** Robin L. Owen, Briony A. Yorke, James A. Gowdy, Arwen R. Pearson

**Affiliations:** a Diamond Light Source Ltd, Diamond House, Harwell Science and Innovation Campus, Didcot, OX11 0DE, UK; b Astbury Centre for Structural Molecular Biology, University of Leeds, Leeds, UK

**Keywords:** macromolecular crystallography, single-crystal microspectrophotometry, radiation damage, myoglobin, cytochrome *c*

## Abstract

The structural information and functional insight obtained from X-ray crystallography can be enhanced by the use of complementary spectroscopies. Here the information that can be obtained from spectroscopic methods commonly used in conjunction with X-ray crystallography and best-practice single-crystal UV-Vis absorption data collection are briefly reviewed. Using data collected with the *in situ* system at the Swiss Light Source, the time and dose scales of low-dose X-ray-induced radiation damage and solvated electron generation in metalloproteins at 100 K are investigated. The effect of dose rate on these scales is also discussed.

## Abstract 2

Data on the rapid reduction of haem proteins in the X-ray beam at synchrotron sources are presented. The use of single-crystal spectroscopy to detect these changes and their implication for diffraction data collection from oxidized species is also discussed.

## 1. Introduction

Structural biology aims to provide detailed insight into biological function at the molecular and atomic level. However, in extrapolating from the static average structures obtained from X-ray crystallography, care must be taken to ensure that radiation damage has not affected the structure determined. While the effect of high absorbed X-ray doses is often readily observed in the form of a loss of diffracted intensity, radiation damage begins to accrue as soon as the X-ray shutter is opened and the absorbed dose is still small (Garman, 2010 ▶). This low-dose damage is not immediately apparent from examination of diffraction images or data processing statistics, but may only be revealed in, for example, a lack of anomalous signal or in the derived electron density map (Holton, 2009 ▶). It is therefore important to assess the time and dose scales over which the initial stages of radiation damage occur. A greater understanding of these, and of the underlying physical processes involved, will aid the design of data collection strategies which result in electron density maps with minimal radiation damage contamination.

On-line single-crystal spectrometers are capable of recording X-ray-induced changes in the crystal during irradiation and have proven invaluable in tracking rapid X-ray-induced changes (Pearson *et al.*, 2004 ▶). The availability of dose-dependent spectral information can guide data collection strategies, enabling the determination of undamaged structures and identification of X-ray-induced intermediates that would have otherwise been impossible to obtain (Berglund *et al.*, 2002 ▶; Schlichting *et al.*, 2000 ▶). Single-crystal spectrometers are now available at synchrotron sources worldwide providing *in situ* access to a range of spectroscopic windows. These include, but are not limited to, the facilities at the European Synchrotron Radiation Facility (ESRF) (Bourgeois *et al.*, 2002 ▶; Royant *et al.*, 2007 ▶; Carpentier *et al.*, 2007 ▶; McGeehan *et al.*, 2009 ▶), National Synchrotron Light Source (NSLS) (Orville *et al.*, 2009 ▶), the on-axis system at the Swiss Light Source (SLS) (Owen *et al.*, 2009 ▶) and the 4DX system at the Advanced Photon Source (APS) (Pearson *et al.*, 2007 ▶). A more detailed recent review of available facilities can be found by Pearson & Owen (2009 ▶).

The role on-line spectroscopy can play in following X-ray-induced changes has proven particularly crucial for metalloproteins (Berglund *et al.*, 2002 ▶; Schlichting *et al.*, 2000 ▶; Yano *et al.*, 2005 ▶; Pearson *et al.*, 2007 ▶; Hough *et al.*, 2008 ▶). Metals are often involved in catalytic, regulatory and structural roles, and precise knowledge of the redox state of a metal in a structure is crucial as redox properties are central to function. During an X-ray experiment, however, metal centres combine the undesirable properties of a high X-ray cross section (photoelectric effect and Compton scattering) and a susceptibility to X-ray-induced change. Spectroscopic studies have revealed the half-life of oxidized species in an X-ray beam to be an order of magnitude less than that of the diffracting power of a crystal, emphasizing the need for complementary methods to monitor these changes. These include [spectroscopic technique used, dose limit (citation)]: disordering of selenomethionine sidechains [XANES, 2 MGy (Holton, 2007 ▶)], reduction of copper nitrate reductase [UV-Vis, XAS, 1.5 MGy (Hough *et al.*, 2008 ▶)], cleavage of anomalous scatterers [XAS, 0.5 MGy (Oliéric *et al.*, 2007 ▶)], photoreduction of putidaredoxin [XAS, 0.3 MGy (Corbett *et al.*, 2007 ▶)] and reduction of Met-Mb [UV-Vis, 0.21 MGy (Beitlich *et al.*, 2007 ▶)].

It is important to note that the optical and X-ray spectroscopies available provide insight into a wide range of physical properties and the spectroscopy to be exploited should be chosen with reference to the problem in hand. UV-Vis absorption, for example, requires a chromophore and provides information on oxidation states and ligand-to-metal charge transfers, while Raman spectroscopy probes the vibrational motions of atoms (Carey, 2006 ▶; McGeehan *et al.*, 2007 ▶; Carpentier *et al.*, 2007 ▶). X-ray spectroscopies such as XANES (X-ray absorption near-edge structure) and EXAFS (extended X-ray absorption fine structure) can provide information on the oxidation state, site symmetry and coordination numbers of metal sites (Yano *et al.*, 2005 ▶; Corbett *et al.*, 2007 ▶; Ellis *et al.*, 2008 ▶).

For proteins not containing a metal or chromophore, the number of spectroscopies that can be exploited for on-line monitoring of X-ray-induced changes is reduced. Vibrational spectroscopies such as Raman and IR absorption are possible, but are not well suited to high-throughput macromolecular crystallography owing to either long acquisition times (Raman) or water absorption (IR). However, in a non-high-throughput environment, single-crystal Raman spectra of very high quality can be obtained and these have proved to be extremely informative, both in identifying the nature of bound ligands or adducts and in identifying radiation-induced changes (Carey, 2006 ▶; McGeehan *et al.*, 2007 ▶; Katona *et al.*, 2007 ▶).

Here we present a study of the effect of dose rate on haem-centre X-ray reduction in myoglobin and cytochrome *c* at cryo-temperatures (100 K). To aid and encourage researchers wishing to combine X-ray crystallography and spectroscopy, we have also included a section with some guidelines on the collection of high-quality artefact-free single-crystal UV-vis spectra (§2).

## 2. Collection of high-quality single-crystal UV-Vis absorption spectra

The use of UV-Vis absorption spectroscopy to establish the radiation sensitivity of a sample is dependent on the collection of high-quality artefact-free spectra. Several key points for the collection of such data are outlined below. Additional useful information on sample preparation and recording of spectra can be found by Wilmot *et al.* (2002 ▶).

**Figure 1:**
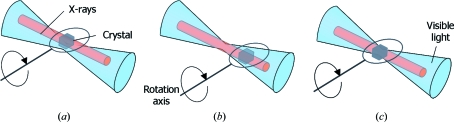
Schematic of an on-axis system showing (*a*) sample rotation axis and X-rays well aligned with respect to one another but misaligned with respect to visible light, (*b*) X-rays and visible light well aligned but sample rotation axis misaligned, and (*c*) good alignment of all components.

Before the collection of spectroscopic data it is important to confirm that both the X-ray beam and the UV-Vis light path probe the same sample volume and intersect exactly with the sample rotation axis (Fig. 1 ▶). Alignment of the light path and rotation axis is best achieved with a small pinhole (∼10 µm diameter) carefully centred at the sample position. The amount of light transmitted by this hole (from both objectives) should be maximized *via* small adjustments to the position of each lens. If an on-axis geometry is used, a scintillator placed at the sample position can be used to confirm alignment of the X-ray and optical light paths [Fig. 5 of Owen *et al.* (2009 ▶)]. Alignment can be confirmed by the use of a standard sample such as a haem protein which exhibits distinctive and easily observable spectral changes upon reduction by X-ray irradiation and of which there are many examples in the literature.

**Figure 2:**
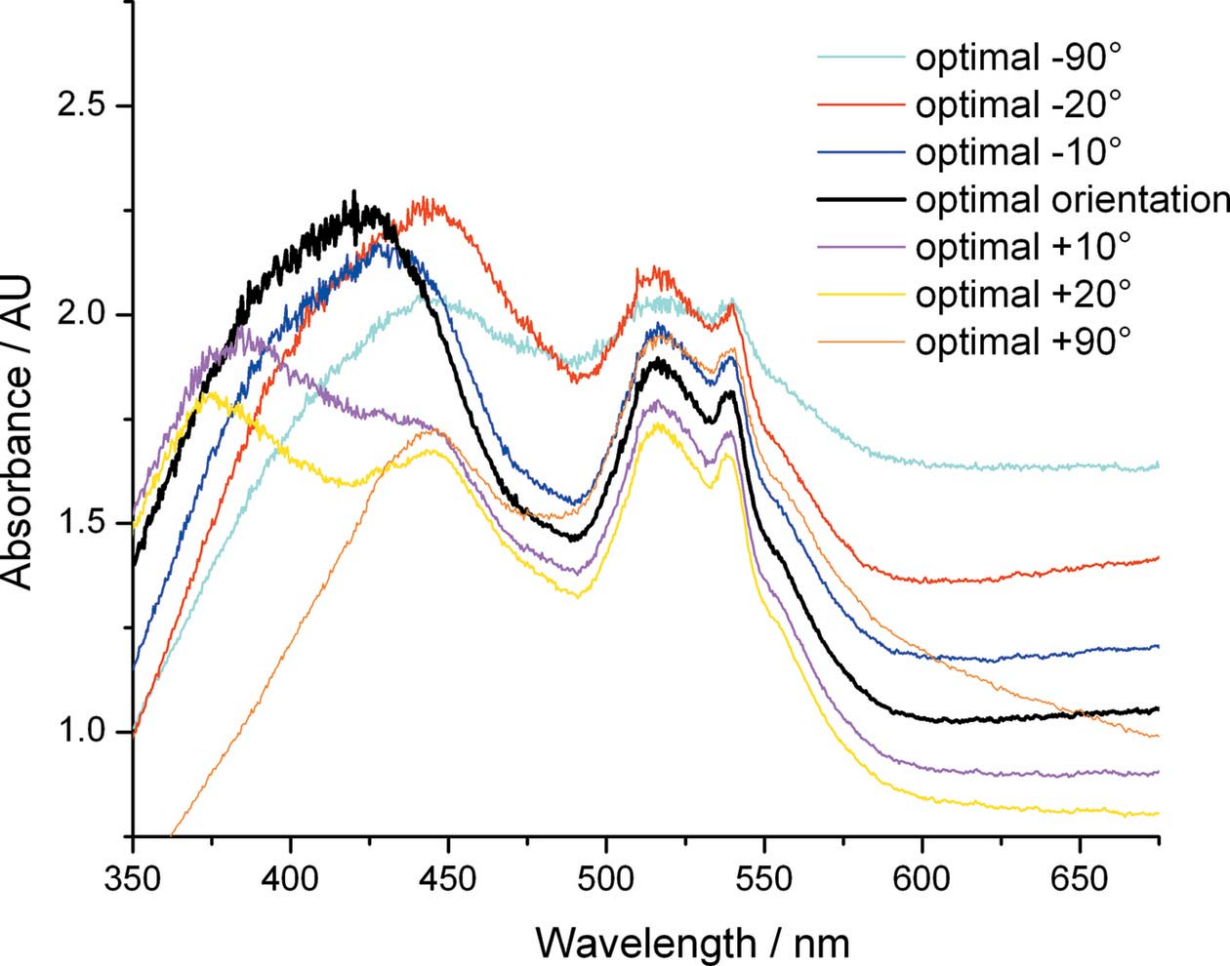
Horse heart cytochrome *c* single-crystal spectra showing variations in both absorption peaks and baseline as a function of crystal orientation. The optimal orientation is obtained when the loop face is almost perpendicular to the UV-Vis light path and a facet of the crystal is in the same plane. Spectra are the average of ten 20 ms exposures and were collected using a 4DX single-crystal spectrometer (4DX Systems), illuminated by a balanced deuterium/halogen light source (Ocean Optics), attached to a Shamrock 163 spectrograph and Newton CCD detector (Andor Technology). For crystallization conditions, see §3.1.

Many ideal practices in sample preparation from X-ray diffraction also apply to single-crystal UV-Vis absorption spectroscopy. The amount of solvent surrounding the crystal should be minimized, *i.e.* crystals should be suspended within a thin film rather than a large volume of solvent and cryoprotectant. Optimized cryoprotection is also critical to a much greater extent than in the X-ray diffraction experiment. Ice is highly undesirable as this reduces the transparency of the sample and leads to aberrant absorption features. Surface ice can be removed, or avoided by careful orientation of the sample though this may then limit the number of suitable positions for data collection. Small changes in position over the surface of the crystal, together with rotation of the sample, can result in large spectral changes both in the form of resolution of features and a changing background level (Fig. 2 ▶). This is due to a combination of prism effects and the anisotropic nature of chromophores in single crystals where they are preferentially oriented by their packing in the lattice (Wilmot *et al.*, 2002 ▶). An optimal orientation that closely resembles the solution spectrum can usually be found. Owing to this dependence on position and angle, if spectral changes are to be tracked during diffraction data collection then an optimal angle must be found and returned to for each UV-Vis data collection. For this reason, in particular at room temperature, it may be necessary to establish the time scales of X-ray-induced changes on a stationary sacrificial crystal prior to diffraction data collection. The accessible rotation range for UV-Vis data collection is also limited by scattering from the loop used for crystal mounting. Loops introduce unwanted spectral features and noise (Fig. 2 ▶) and should therefore be chosen so that the focal spot of the lamp comfortably fits within the loop cross-sectional area. An ideally mounted sample will also provide sufficient space for a reference spectrum to be collected on the mother liquor or cryobuffer alone. This is particularly advantageous for crystals of high optical density as longer exposure times can be used before lamp features saturate the detector introducing spectral artefacts.

While it is difficult, or almost impossible, to improve the spectral properties of an inherently poor or icy sample, weak spectra or spectra from crystals with a high optical density can be improved at the point of data collection by careful choice of data collection parameters. For example, the emission spectrum of the xenon light source used in the SLS microspectrophotometer is considerably stronger at longer wavelengths (>500 nm) than in the 350–450 nm region. This has the consequence that, when recording a spectrum from a sample with high optical density at low wavelength, *e.g.* haem, increasing the exposure time in order to produce a measurable change in absorbance for the strongly absorbing Soret band (usually located between ∼400 and 450 nm) results in saturation of the detector above 500 nm, obliterating the weaker α- and β-bands (located between 500 and 600 nm). Filters can be used to equalize the illumination spectrum to avoid this problem.

## 3. Materials and methods

### 3.1. Sample preparation

Solutions of horse myoglobin (Sigma) at 20 mg ml^−1^ in 50% (*v*/*v*) 2-methyl-2,4-pentanediol (MPD) were doped with increasing amounts of sodium bromide (0–1.0 *M* in 0.1 *M* steps), and solutions of bovine cytochrome *c* (Sigma) at 20 mg ml^−1^ in 50% (*v*/*v*) MPD were doped with NaCl (0–1.0 *M* in 0.1 *M* steps). To avoid any confounding elements in this study of the effects of X-ray illumination, no additional buffer components were added. The solutions were prepared from stocks such that the concentration of protein was constant in all samples. Thin films of the solutions were cryo-cooled in 0.2–0.5 mm-diameter nylon (20 µm thick) loops at 100 K using a cryojet (Oxford Instruments). Visual inspection indicated the film thickness to be less than the thickness of the loops.

Horse cytochrome *c* (Sigma) crystals were grown according to the method of Sanishvili *et al.* (1994 ▶), using drops containing 40 mg ml^−1^ cytochrome *c*, 50 m*M* potassium phosphate pH 6.8 and 22% PEG 1000 (*w*/*v*) equilibrated against a reservoir of 30% (*w*/*v*) PEG 1000 and 50 m*M* potassium phosphate pH 6.8. Crystals (∼100 × 100 × 300 µm) were soaked for 2 min in mother liquor containing increasing amounts of NaCl (0–0.6 *M*) or Na_2_MoO_4_ (0–0.6 *M*), harvested in nylon loops and directly cryo-cooled at 100 K in a cryojet without any additional cryoprotectant.

### 3.2. Spectroscopic data collection

The on-axis arrangement previously described (Owen *et al.*, 2009 ▶) at beamline X10SA of the SLS was used for all X-ray reduction experiments. An X-ray beamsize of 50 × 50 µm was used for all experiments, while the focal spot size of the Xenon lamp at the sample was ∼35 µm.

**Figure 3:**
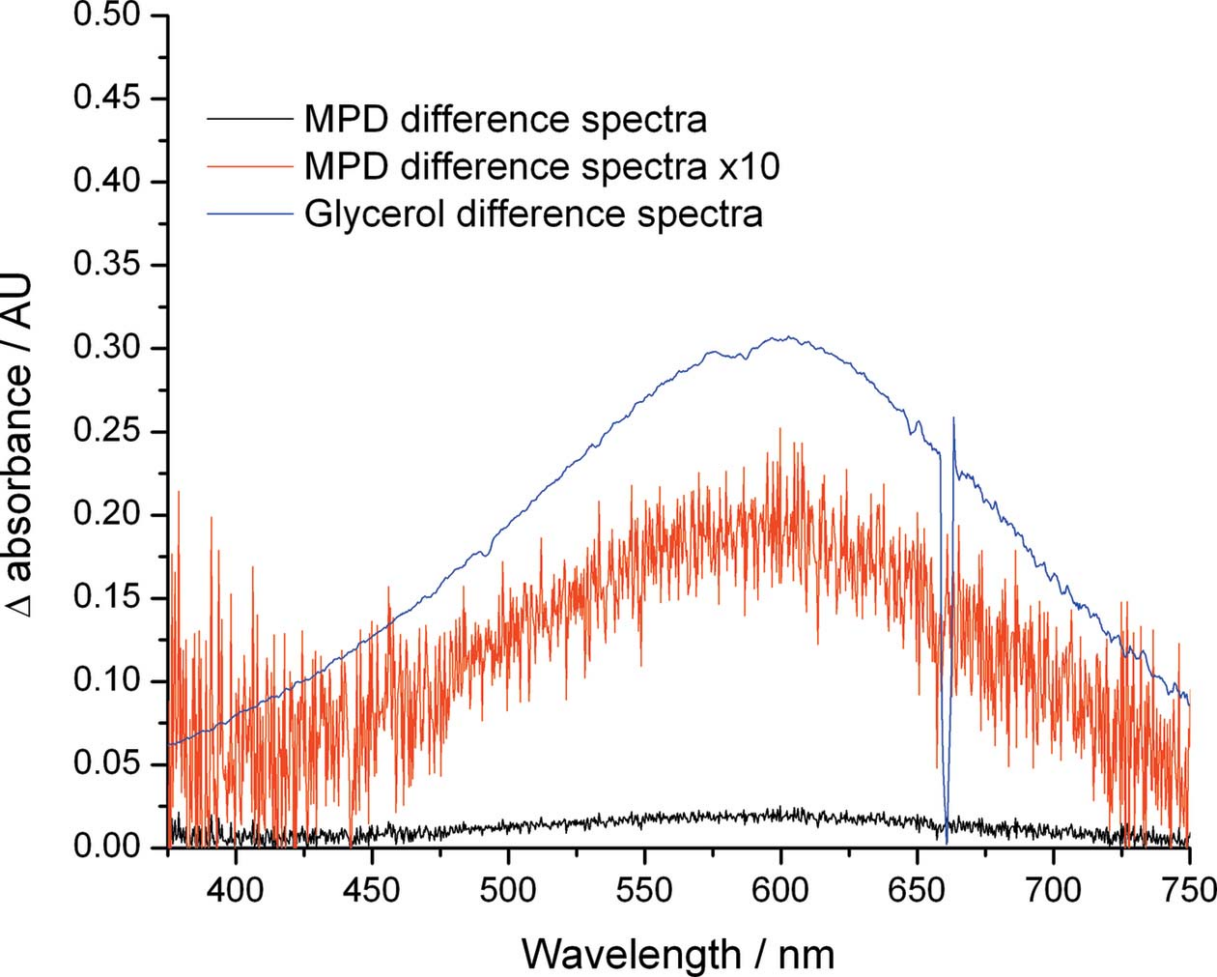
Difference spectra (X-ray shutter open − X-ray shutter closed) showing the appearance of a broad feature centred at 600 nm in solutions of 50% (*v*/*v*) MPD (recorded in this study) and 40% (*v*/*v*) glycerol [recorded at the ESRF (McGeehan *et al.*, 2009 ▶)] at 100 K during X-ray exposure. The discontinuity in the glycerol spectrum at 680 nm is a deuterium lamp artefact.

When illuminated by X-rays, glycerol has been reported to give a broad strong visible absorption signal centred at 600 nm, that has been assigned to the generation of solvated electrons (McGeehan *et al.*, 2009 ▶). As myoglobin and cytochrome *c* have absorption bands that extend into the red and near-IR regions [*i.e.* the α and β bands are in the 500–600 nm region (Lippard & Berg, 1994 ▶)], we investigated MPD as an alternative cryoprotectant for the thin-film studies. It has been reported that there is no significant change in MPD absorption over the spectral range of interest upon X-ray irradiation (McGeehan *et al.*, 2009 ▶) (Fig. 3 ▶). On-line UV-Vis spectra were recorded over 30 s (53.6 spectra per second with an acquisition time of 10 ms per spectrum) and the X-ray shutter was opened a short time (<4 s) after the UV-Vis spectra accumulation began (X-ray energy 12.4 keV with a flux of 1.1 × 10^11^ photons s^−1^), allowing time courses to be fitted over >25 s of X-ray exposure.

In order to probe the effect of changing the mother liquor X-ray absorption cross section on the rate of haem centre reduction, on-line UV-Vis spectra were recorded, as described above, from myoglobin and cytochrome *c* thin films in triplicate for each halide (Cl^−^ and Br^−^) concentration during continual X-ray exposure as above. Thin films were used since the optical density of the cytochrome *c* single crystals was so high that changes in the Soret band could not be accurately observed. However, to confirm similar effects were seen in crystals and in solution, the experiment was repeated using cytochrome *c* crystals doped with NaCl and Na_2_MoO_4_, as changes in the α and β bands (but not the Soret) could be directly compared with those observed in the thin films.

Finally, a second series of spectra were recorded for the myoglobin thin films doped with NaBr (0–1 *M*) at a lower flux rate of 5 × 10^10^ photons s^−1^, maintaining the X-ray energy at 12.4 keV.

### 3.3. Data analysis

Single-wavelength time courses for the shift in the Soret band (from 413 to 426 nm for myoglobin and 411 to 415 nm for cytochrome *c*) and the appearance of the α (557 nm myoglobin, 535 nm cytochrome *c*) peaks (characteristic of reduced haem) and at 600 nm (assigned to solvated electron formation) were extracted from the data. Kinetic parameters were determined using *Gnuplot*, *Biokine* (Biologic) and *OriginPro* (Origin Lab) and analysed using *OriginPro*. Myoglobin peaks were assigned based on spectra for myoglobin reduced by ^60^Co γ radiation at 77 K [where myoglobin cannot undergo the conformational rearrangements that normally accompany reduction (Gasyna, 1979 ▶)]. Cytochrome *c* peaks were assigned based on literature values (Keilin & Slater, 1953 ▶).

Absorbed-dose calculations for the protein samples were carried out using *RADDOSE* (Paithankar *et al.*, 2009 ▶), assuming a constant sample volume and a non-rotating sample.

**Figure 4:**
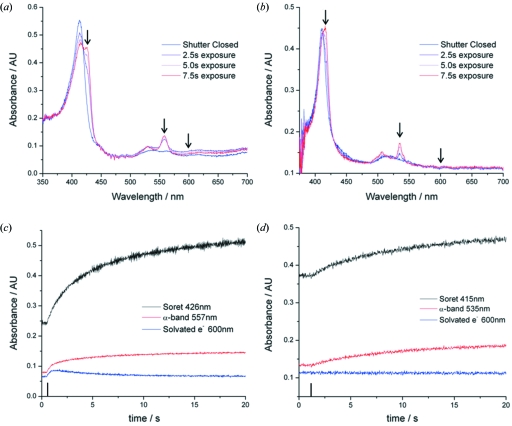
Absorption spectra of (*a*) myoglobin and (*b*) cytochrome *c* thin films doped with 0.5 *M* NaBr before and after exposure to X-ray radiation recorded using the SLS on-axis microspectrophotometer. Several intermediate spectra are shown to highlight the isosbestic point in each system. The arrows show, from left to right, the position of the Soret and α-bands upon X-ray reduction and the 600 nm feature. Sample extracted time courses for (*c*) myoglobin and (*d*) cytochrome *c* showing the changes in absorbance at the three wavelengths of interest *versus* time (accumulated dose). The upright line on the X-axis indicates the point at which the X-ray shutter was opened; from this point the sample was continuously irradiated.

Rate constants for myoglobin and cytochrome *c* reduction by X-rays were determined for two features in the spectra: the shift in the Soret band and the increase in the α-band (Fig. 4 ▶). Also fitted were the time courses for absorbance changes at 600 nm for both proteins in 50% (*v*/*v*) MPD as well as for 50% (*v*/*v*) MPD alone. All data were best fitted with a double exponential, as has previously been reported for met-myoglobin X-ray reduction as a function of incident X-ray energy (Beitlich *et al.*, 2007 ▶). This function is of the form
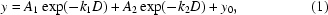
where *y* is the absorbance of the Soret or α-band, *k*_1_ and *k*_2_ are the fast and slow rates, respectively, *A*_1_ and *A*_2_ are the amplitudes of the two exponents, and *D* is the absorbed dose. All rate constants were thus in units of dose^−1^. The function is constrained so that the fast process described by *k*_1_ occurs first.

## 4. Results

### 4.1. Effect of cryoprotectant used on spectral changes

In order to establish marker wavelengths representative of solvated electrons rather than of long-wavelength haem protein bands, optical spectra were collected from the cryoprotectant, MPD, alone during X-ray illumination (Fig. 3 ▶). These data are compared with spectra from 40% glycerol previously recorded at the ESRF and reported by McGeehan *et al.* (2009 ▶).

Although it has been reported that no distinct absorbance peak associated with solvated electrons is observed in solutions of MPD (McGeehan *et al.*, 2009 ▶), a weak broad increase in absorbance in this region was observed when 50% (*v*/*v*) MPD alone was exposed to X-rays. The change in absorbance of MPD at 600 nm is small, but is easily resolvable by the SLS microspectrophotometer.

Since the absorbance change at 600 nm upon X-ray illumination of MPD is so small, it does not mask the temporal evolution of haem protein α and β bands between 500 and 560 nm. MPD was therefore used as a cryoprotectant for the subsequent experiments reported here.

### 4.2. X-ray-induced changes to oxidized myoglobin and cytochrome *c*

To establish whether the rate of reduction of the haem centres of myoglobin and cytochrome *c* during X-ray irradiation is affected by X-ray dose rate at 100 K, in contrast to ‘classical’ radiation damage (loss of diffracted intensities, changes in unit-cell dimensions, disulfide bond breakages, decarboxylations, *etc.*) which is dose-rate independent at cryo-temperatures (Garman, 2010 ▶), we monitored the rate of haem reduction during continuous X-ray exposure in cryo-cooled thin films (∼20 µm thick) of both proteins. To alter the dose rate we doped solutions of both proteins with increasing concentrations of NaBr and NaCl. An increase in NaBr concentration from 0 *M* to 1 *M*, for example, results in a 49% increase in the X-ray absorption cross section of the solution, with a consequent change in absorbed dose from 19 kGy s^−1^ to 28 kGy s^−1^ (for the same incident flux and exposure time, an increase in NaCl concentration from 0 *M* to 1 *M* corresponds to a change in absorbed dose from 16 to 26 kGy s^−1^). We also prepared cryo-cooled cytochrome *c* crystals that had been soaked in artificial mother liquor containing NaCl, NaBr and Na_2_MoO_4_.

**Figure 5:**
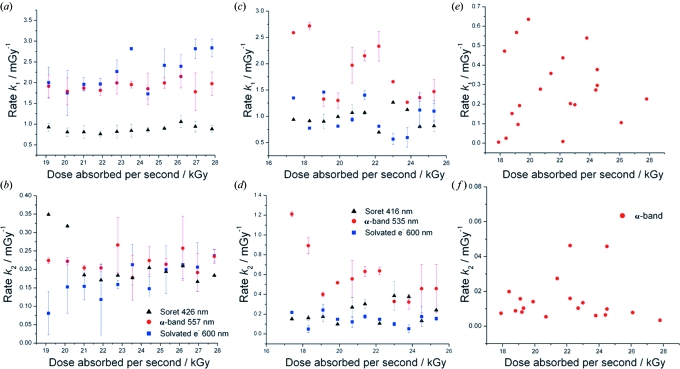
Rate constants for (*a*, *b*) myoglobin thin films doped with increasing concentrations of NaBr (0–1.0 *M* in 0.1 *M* steps from left to right), (*c*, *d*) cytochrome *c* thin films doped with increasing concentration of NaCl and (*e*, *f*) horse heart cytochrome *c* crystals doped with NaBr, NaCl and Na_2_MoO_4_. *k*_1_ for each is shown in the top row, while *k*_2_ is shown at the bottom. Each point is the mean value of three observations. Cytochrome *c* crystal *k*_2_ data have been scaled to exclude a single outlier at 0.23 mGy^−1^. Owing to saturation effects and the difficulty in tracking the weak solvated electron signal in crystals, only the α-band has been tracked for cytochrome *c* crystal data.

The spectral changes that occur upon X-ray irradiation of both proteins are indicative of haem reduction. The Soret band is red shifted and the broad band at 530–550 nm splits into two distinct peaks [Figs. 4(*a*) and 4(*b*) ▶]. A clear isosbestic point (indicating a two-state system) at 419.5 nm and 413.1 nm was observed in the thin-film data for myoglobin and cytochrome *c*, respectively. Despite the presence of an isosbestic point the time courses extracted for the shift in the Soret band and the appearance of the α-band, as well as the spectral changes at 600 nm, show a biphasic behaviour in all samples [as previously observed by Beitlich *et al.* (2007 ▶) for myoglobin] indicating that two kinetically distinct processes are occurring [Figs. 4(*c*) and 4(*d*) ▶]. The changes at 600 nm, shown to be the λ_max_ of solvated electrons in MPD (§4.1), were taken to be representative of solvated electron formation and decay. The extracted spectral time courses were best fit by a double exponential function and the results are summarized in Fig. 5 ▶.

The observed rate constants *k*_1_ and *k*_2_ (Gy^−1^) are each similar at all three wavelengths studied and are essentially constant with absorbed dose, indicating that the X-ray-induced rapid reduction of the haem centres and the rate of solvated electron generation is independent of dose rate.

**Figure 6:**
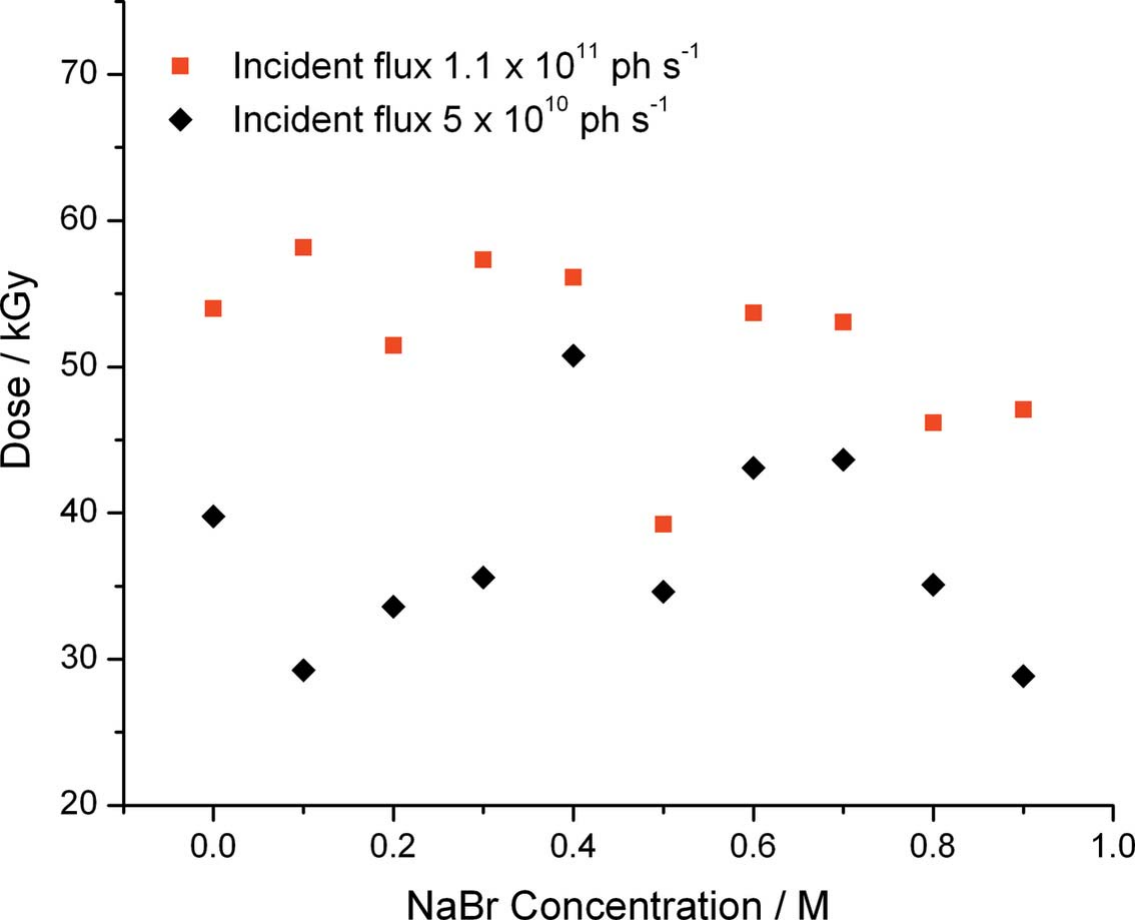
The absorbed dose at which the solvated electron population reaches a maximum in thin films of myoglobin (as determined from the change in absorption at 600 nm) as a function of both sodium bromide concentration and incident flux rate. Each point is the average of three measurements.

The dose at which the population of solvated electrons reaches a maximum was determined from the fit parameters *A*_1_, *k*_1_, *A*_2_ and *k*_2_ for the myoglobin thin film 600 nm time courses at two incident fluxes and a range of NaBr concentrations by differentiation of equation (1) (Fig. 6 ▶). This ‘turnover dose’ was found to be slightly increased in the high-flux regime, but almost constant at different halide concentrations (mean value 45 kGy).

## 5. Discussion

Extensive studies of radiation damage by several groups have demonstrated that classical radiation damage (loss of diffracted intensities, changes in unit-cell dimensions, disulfide bond breakages, decarboxylations, *etc.*) is purely a function of the cumulative absorbed dose (Holton, 2009 ▶; Garman, 2010 ▶). The cumulative absorbed dose is dependent on the X-ray absorption cross section of the illuminated volume (which can include the crystal itself as well as surrounding mother liquor and the mount). A comprehensive study by Kmetko *et al.* (2006 ▶) showed clearly that the composition of the mother liquor, and hence the absorption cross section of the sample, does affect the rate of accumulation of classical radiation damage, and suggested that attempts to reduce the X-ray absorption cross section of the mother liquor should extend crystal life-time at 100 K because the dose is reduced. 

Here we have investigated whether the rapid X-ray reduction of the haem centre in myoglobin and cytochrome *c* at low cumulative X-ray doses is also independent of dose rate. Our data show clearly that the rate of accumulation of this low-dose damage is independent of dose rate at 100 K.

A relatively small increase and subsequent decrease in absorbance was observed at 600 nm upon irradiation of myoglobin, cytochrome *c* and also of MPD alone. By comparison with the better characterized absorption maximum of glycerol upon X-ray irradiation (Ershov & Pikaev, 1968 ▶; McGeehan *et al.*, 2009 ▶), this was attributed to solvated electrons. Interestingly this peak grows and decays with rate constants that are strikingly similar to the two rate constants required to fit the X-ray-driven reduction of the haem proteins reported here. Beitlich *et al.* (2007 ▶), who studied changes at 557 nm (nominally the α band of myoglobin, but where the contribution of the absorbance of solvated electrons can also be significant) upon X-irradiation of myoglobin, suggested that *k*_1_ (the faster rate) was representative of the formation of solvated electrons upon X-ray illumination. They assigned the slower *k*_2_ as representative of the haem reduction rate when the concentration of solvated electrons had reached a steady state. The similarity of the *k*_2_ for both myoglobin and cytochrome *c* reduction to that we observed for the decay of the 600 nm feature, which presumably reflects the decay to a steady-state level of solvated electrons, supports this hypothesis.

In view of this, we determined the dose at which the population of solvated electrons reaches a maximum (*i.e.* the maximal absorbance at 600 nm), before decaying to its steady state and found that this occurs at ∼45 kGy. This value is a function only of the absorbed dose and not the mother liquor composition (other than in the way this composition affects the absorption cross sections) or incident flux. While this dose is somewhat smaller than the dose limits summarized in §1 for the reduction of metal centres at 100 K by synchrotron X-ray irradiation (0.2–2 MGy), it is comparable with dose limits observed spectroscopically during γ-radiolysis of myoglobin at relatively low dose rates [yield of 50% reduced myoglobin generated at an absorbed dose of 20 kGy with an incident dose rate of 220 Gy min^−1^ (Denisov *et al.*, 2007 ▶)].

Taken together, these observations suggest that reducing the absorption cross section of the crystallization mother liquor, either by transferring the crystal into a weakly absorbing mother liquor/cryoprotectant solution or by backsoaking to remove non-bound anomalous scatterers, will somewhat extend the lifetime of oxidized redox centres in the X-ray beam. However, this gain is likely to be small, as the rate of the haem centre reduction appears to be strongly coupled to the generation of solvated electrons that, in myoglobin in MPD, reaches a maximum at an absorbed dose of only 45 kGy. With the beamline fluxes available at third-generation synchrotron sources, this can easily be reached within the time required to collect a single diffraction image.

Therefore, a composite data collection strategy in which small wedges of data are collected from several crystals (or several points on a large single crystal) remains the best, and possibly the only, approach to obtaining the structures of fully oxidized and partially oxidized species.

## 6. Summary

Complementary methods provide valuable information both on the properties of a sample and how these change upon X-ray irradiation. These changes occur on dose scales that are much smaller than are observable through changes in the quality of the X-ray diffraction pattern. The maximum population of solvated photoelectrons in this study was reached by a dose of ∼45 kGy, some three orders of magnitude smaller than the dose limit based on diffracting power (Owen *et al.*, 2006 ▶). At third-generation synchrotron sources, absorbed doses of this magnitude can be easily reached when collecting a single image, highlighting the limitations of diffraction-based methods for tracking low-dose radiation damage. The use of complementary methods during the diffraction experiment is therefore extremely important to establish what structural state the recorded diffraction data describe, and is essential for the recognition and tracking of low-dose radiation damage.
